# Uterine intravenous leiomyomatosis with an isolated large metastasis to the right atrium: a case report

**DOI:** 10.1186/s13000-019-0913-2

**Published:** 2020-01-11

**Authors:** Mitsutake Yano, Tomomi Katoh, Yoshie Nakajima, Shiro Iwanaga, Rei Kin, Eito Kozawa, Masanori Yasuda

**Affiliations:** 1 Department of Pathology, Saitama Medical, University International Medical Center, 1397-1 Yamane, Hidaka-City, Saitama 350-1298 Japan; 2 Department of Obstetrics and Gynecology, Oita University Faculty of Medicine, 1-1 Idaigaoka, Hasama-machi, Yufu-shi, Oita, 879-5593 Japan; 3 Department of Cardiology, Saitama Medical University International Medical Center, 1397-1 Yamane, Hidaka-City, Saitama 350-1298 Japan; 4 Department of Pathology, Saitama Medical University Hospital, 38 Morohongo, Iruma-Gun, Moroyama, Saitama 350-0495 Japan; 5 Department of Diagnostic Radiology, Saitama Medical University Hospital, 38 Morohongo, Iruma-Gun, Moroyama, Saitama 350-0495 Japan

**Keywords:** Uterus, Intravenous leiomyomatosis, Cardiac metastasis, Right atrium, Case report

## Abstract

**Background:**

An intravenous leiomyomatosis is a special type of uterine leiomyoma characterized by the formation of benign leiomyomatous tissue within the vascular wall. Although histologically benign, intracardiac metastasis can lead to circulatory failure, and death, if untreated. Herein, we report on a case of a uterine intravenous leiomyomatosis with an isolated large adherent metastasis in the right atrium of the heart.

**Case Presentation:**

A 52-year-old Japanese woman sought medical attention at our hospital for lower abdominal pain. A 27-cm uterine mass was detected on clinical imaging, with a 78 × 47-mm mass in the right atrium detected on preoperative echocardiography. Intracardiac mass resection and tricuspid annuloplasty were performed as the first-stage surgery. The pedicle of the tumor was adherent to the wall of the atrium. On histological examination, the tumor was found to consist of spindle-shaped cells with eosinophilic cytoplasm, without atypia, but with a myxoid change, and rich microvascularization of the pedicle. Total abdominal hysterectomy was performed as the second-stage surgery, with confirmation of the diagnosis as uterine intravenous leiomyomatosis with an isolated metastasic lesion to the right atrium. There has been no evidence of tumor recurrence in the 15 months since surgery.

**Conclusion:**

We report a unique case in which a large right atrial leiomyoma was identified following a uterine intravenous leiomyomatosis. Our case exemplifies that intravenous leiomyomatosis metastatic tumors have the potential to grow via their vascularization.

## Background

Intravenous leiomyomatosis (IVL) is a special type of uterine leiomyoma, characterized by the formation of benign leiomyomatous tissue within the vascular vessels of the uterus. The tumor typically grows along vascular vessels and, thus, can extend to the iliac vein, inferior vena cava and, even to the heart. IVL develops in only 0.1% of women with uterine leiomyomas, with intracardiac involvement identified in 10–40% of IVL cases [1]. IVL with intracardiac involvement was first reported in 1907, with fewer than 300 cases presently documented in the literature, since then [2]. Although histologically benign, intracardiac IVL extension can lead to circulatory failure or death if left untreated [3]. The typical features of IVL include contiguous pelvic and intravenous masses, with sausage-shaped lesions in the inferior vena cava, whereas intracardiac tumors arising from IVL typically show a worm-like appearance [4]. Some studies have reported on isolated intracardiac tumor from uterine IVL that are adherent to the cardiovascular wall [5–8]. Herein, we report such a case of uterine IVL that clinicopathologically preceded IVL metastasis into the right atrium.

## Case presentation

The patient provided consent for publication of this case report.

### Clinical history

**Fig. 1:**
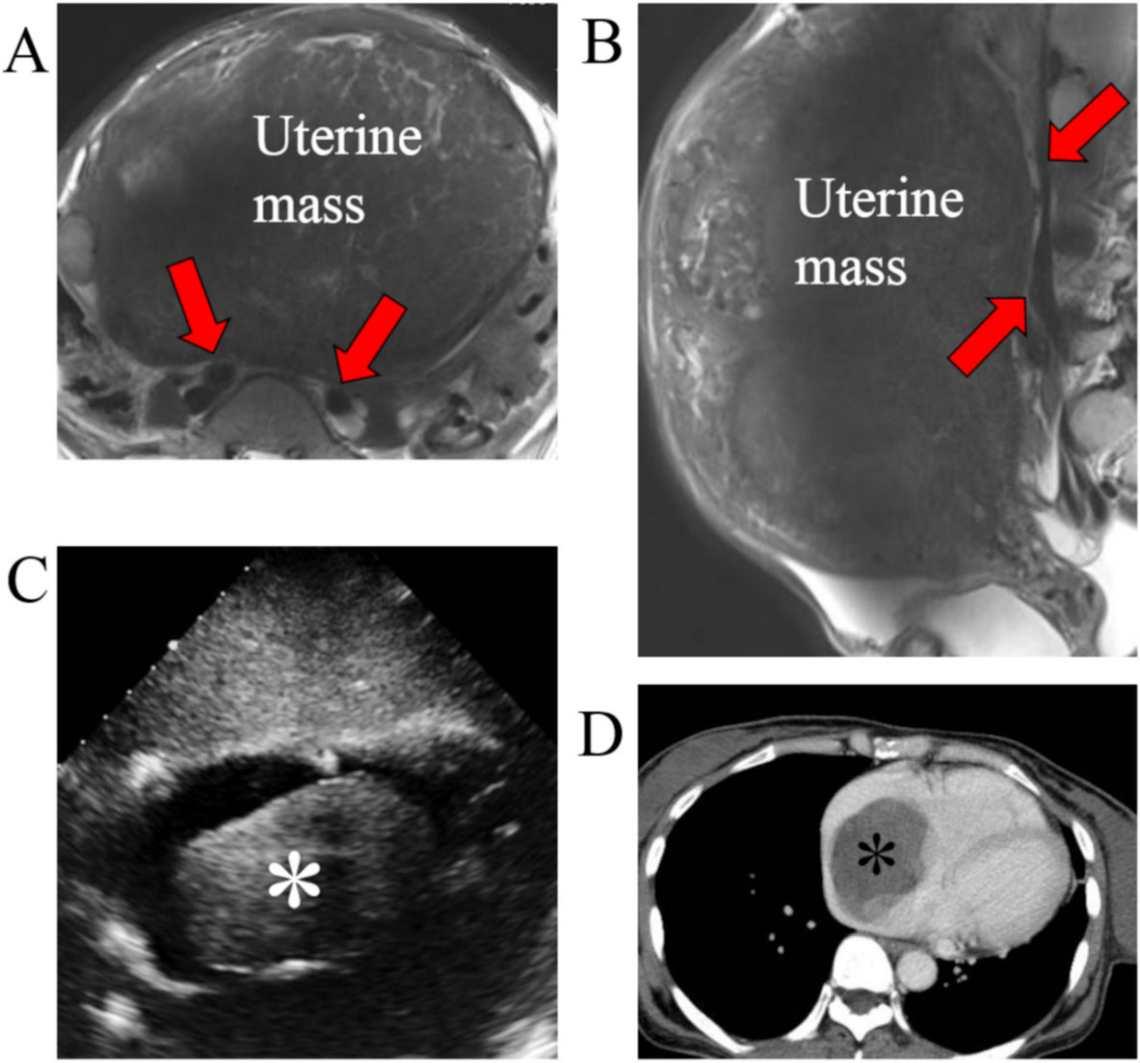
Clinical imaging findings: **a** and **b** T2-weighted MRI revealed a 27-cm mass in the uterus. No tumor was detected in the iliac vein (A, red arrows) or in the inferior vena cava (B, red arrows). **c** Echocardiography imaging revealed a 78 × 47-mm mass (asterisk) in the right atrium. **d** Enhanced computed tomography imaging confirmed a 75-mm mass (asterisk) in the right atrium

The patient was a 52-year-old Japanese woman, gravida 2, para 2, who sought medical attention at our hospital because of lower abdominal pain. The patient had a past history of uterine leiomyoma and duodenal ulcer, the latter having been managed conservatively with medication. A huge uterine mass was detected by abdominal computed tomography (CT). Magnetic resonance imaging (MRI) revealed the presence of a 27-cm mass in the uterus, indicative of a leiomyoma (Fig. 1a). No tumor was detected in the inferior vena cava, iliac vein, or ovarian vein (Fig. 1a, b). The patient was scheduled for surgery for removal of the uterine tumor. However, during routine preoperative follow-up, a 78 × 47-mm mass was identified in the right atrium of her heart on echocardiography (Fig. 1c). Other features of the echocardiography included a respiratory variation of the diameter of the inferior vena cava of 12 mm, and a left ventricular ejection fraction of 64%, both of which were within normal range. Systemic enhanced CT revealed a 75-mm mass in the right atrium (Fig. 1d), with no evidence of lung metastases. Under a preoperative diagnosis of metastatic lesion or cardiac myxoid tumor, intracardiac mass resection and tricuspid annuloplasty were performed as the first-stage surgery. After surgery for the cardiac mass, gonadotropin releasing hormone agonist therapy was administered for 6 months for the management of the uterine mass. With no change in the tumor size, total abdominal hysterectomy was performed as a second-stage surgery. The diagnosis was confirmed as uterine IVL, with an isolated metastasis to the right atrium. There has been no recurrence of the tumor over the period of 15 months following surgery, with no requirement for anti-estrogen therapy during this period.

### Gross and microscopic findings

**Fig. 2:**
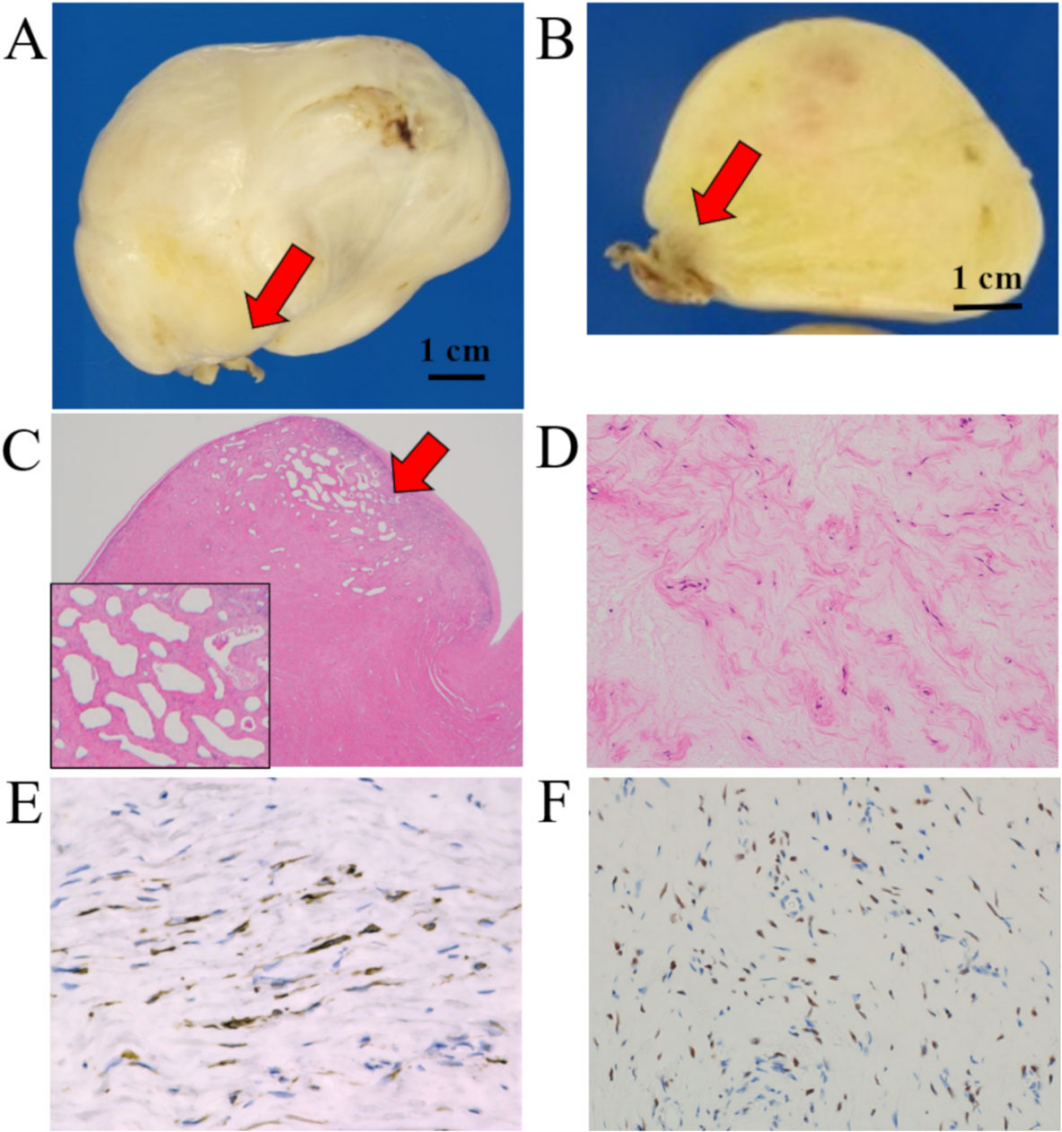
The macroscopic and microscopic findings and immunohistochemical staining of the intracardiac tumor: **a** and **b** Macroscopically, the tumor was a 78 × 58 × 55-mm mass, with a pedicle (red arrows) clinging to the right atrium. **b** The cut surface showed a gray-white colored solid mass. **c** and **d** Microscopically, the rich microvasculature of the pedicle was shown (red arrows); **d** The tumor consisted of spindle-shaped cells, with eosinophilic cytoplasm, lacked atypia, and was accompanied by a myxoid change (high power view). **e** and **f** Immunohistochemically, the tumor cells were positive for (**e**) desmin and (**f**) ER

An ovoid, gray-white colored mass, with a tough texture, was removed (78 × 58 × 55 mm in size). The mass was adherent to the wall of the right atrium via a pedicle (Fig. 2a, b). Histologically, the tumor consisted of spindle-shaped cells, with eosinophilic cytoplasm, lacked atypia, and was accompanied by a myxoid change. The pedicle adhering to the right atrium was richly microvascularized (Fig. 2c, d). Immunohistochemical staining was positive for α-smooth muscle actin (α-SMA), desmin (focal), and estrogen receptor (ER), but negative for progesterone receptor (PgR) and CD10 (Fig. 2e, f).

**Fig. 3:**
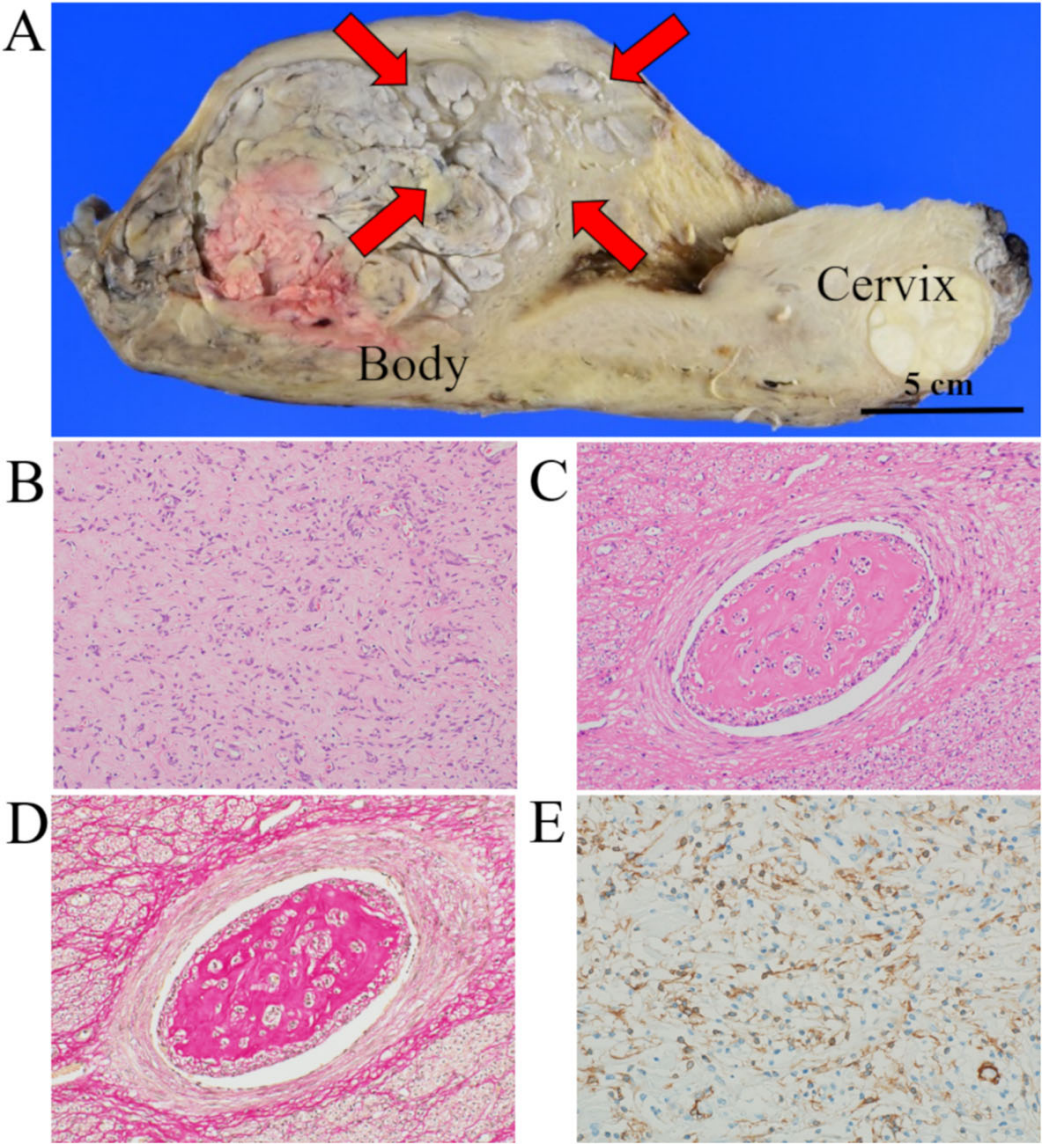
The macroscopic and microscopic findings and immunohistochemical staining of the uterine mass: **a** Macroscopically, the cut surface presented as a gray-white colored mass, with a “worm-like” appearance (red arrows). **b** Microscopically, the uterine large myoma presented spindle-like smooth muscle cells without atypia and mitosis. **c** and **d** The tumor showed a growth of benign leiomyomatous tissues within vascular vessels (C, H&E; D, Elastica van Gieson). **e** Immunohistochemically, the tumor was positive for α-SMA

Macroscopically, the uterus was enlarged and the cut surface showed a white solid mass with a “worm-like” appearance (Fig. 3a). Histopathologic examination revealed multiple benign leiomyomatous tissues within the vascular vessels (Fig. 3b, c). Immunohistochemical stains were positive for α-SMA, desmin (focal), ER, and PgR, but negative for CD10 (Fig. 3d, e). Peritoneal cytology was negative for malignancy.

## Discussion and conclusions

IVL has the potential to grow along blood vessels, extending to the iliac vein, inferior vena cava, and even to the heart. Typically with IVL, the pelvic and intravenous masses are continuous and the intravenous lesions do not invade or adhere to the vessel wall [4].

In the present case, however, the intracardiac metastasis arising from the uterine IVL was isolated and was adherent to the endocardium of the atrium, with no evidence of tumor occurrence in the inferior vena cava, internal iliac vein or ovarian vein. The tumor of the right atrium was an independent metastasis of the uterine IVL, mimicking a primary cardiac myxoma. In the presence of uterine IVL, it is necessary to distinguish the nature of the intracardiac mass, regardless of the absence of its continuity with the uterus. Of note, the intracardiac tumor in the present case was larger than the diameter of the inferior vena cava and, thus, was considered to have developed within the heart. This differs from the report by Maneyama et al. [9] in which a spontaneous migration of a residual IVL to the heart, via the blood stream after hysterectomy, was described. The intracardiac tumor of the present case was adherent to the wall of the right atrium wall via a richly vascularized pedicle. This microvasculature is what likely allowed the tumor to grow.

A summary of previous reports [5–8] on the occurrence of an isolated cardiac metastasis from uterine IVL is provided in Table 1. The tumor diameter of the present case was more than twice that of previously reported cases. The intracardiac mass was identified after hysterectomy for IVL in 3 of 5 cases, with uterine fibroids identified in the other 2 cases. Of note, in all previously reported cases, there was concurrent evidence of lung metastases, which was not the case for our patient. Tumor progression after surgery was not identified in any of these previous cases. Ordulu et al. [10] reported the expression of HMGA2 protein, a driver for tumor metastasis [11], in 58% of IVL cases, which is higher than the 32% in cases with typical uterine leiomyoma. Regional chromosomal alterations of variable frequencies were also observed in IVL, showing overlaps with uterine leiomyosarcoma [12]. Thus, IVL has an intermediate biological propensity between a benign and malignant status. Recurrence of IVL tends to occur in younger patients. Du et al. [1] have suggested that young patients should be treated using hysterectomy and salpingo-oophorectomy if the patient does not wish to maintain fertility. Mizoguchi et al. [13] pointed out that anti-estrogen therapy may be an effective treatment if the patient has not yet entered menopause. These results suggest that IVL treatment requires tumor reduction and blockage of blood flow and estrogen to limit continued growth of the tumor and possible metastasis.

**Table 1:** Cardiac metastasis of intravenous leiomyomatosis

Case (year)	Age (years)	Past history	Symptoms	Size (mm)	Adhere	Metastasis	Treatment	Prognosis (follow-up)
Present case (2018)	52	Uterine leiomyoma	Abdominal pain	78	Anterior wall of RA	Heart	TR + TH + TVP	PFS
							+GnRHa	(14 months)
Thukkani et al., (2005) [5]	36	TH + USO for IVL	Abdominal swelling	15	TV	Heart, lung	TR + USO + TVP	PFS
		(at 26 years)					+GnRHa	(12 months)
Baboci et al., (2014) [7]	51	TH for IVL	Shortness of breath	NA	Anterior wall of RA,	Heart, lung	TR + lung	PFS
		(at 47 years)			TV, chordae tendineae		segmentectomy	(2 years)
Lin and Liu, (2014) [8]	43	TH + BSO for IVL	Palpitation,	15	TV	Heart, lung	None	PFS
		(at 42 years)	chest distress					(7 years)
Zhang and Lang, (2016) [6]	40	Uterine mass	None	30	Chordae tendineae,	Heart, lung	TR + TH + BSO	PFS
					papillary muscles		+TVP + GnRHa	(2 months)

*RA* Right atrium, *TR* Tumor resection, *TH* Total hysterectomy, *TVP* Tricuspid valvuloplasty; *GnRHa* Gonadotropin releasing hormone agonist, *PFS* Progression free survival, *USO* Unilateral salpingo-oophorectomy, *IVL* Intravenous leiomyomatosis, *TV* Tricuspid valve, *NA* Not available, *BSO* Bilateral salpingo-oophorectomy

In conclusion, we report a unique case in which a right atrial leiomyoma was identified following a uterine leiomyoma. The uterine IVL had an isolated large metastasis to the right atrium that was adherent to the endocardium via a richly vascularized pedicle. Our case exemplifies that IVL metastatic tumors have the potential to grow via their vascularization.

## Data Availability

All data generated or analyzed during this study are included in this published article.

## Abbreviations

CT: Computed tomography
ER: Estrogen receptor
IVL: Intravenous leiomyomatosis
MRI: Magnetic resonance imaging
PgR: Progesterone receptor
α-SMA: α-smooth muscle actin
